# Nurses’ relevance in a century of tuberculosis prevention actions in Brazil

**DOI:** 10.1590/0034-7167-2025-0401

**Published:** 2025-12-08

**Authors:** Fernanda Dockhorn Costa Johansen, Maria do Socorro Nantua Evangelista, Maria Rita Bertolozzi, Ethel Leonor Noia Maciel

**Affiliations:** I Ministério da Saúde, Coordenação-Geral de Vigilância da Tuberculose, Micoses Endêmicas e Micobactérias não Tuberculosas. Brasília, DF, Brazil; II Universidade de São Paulo. São Paulo, SP, Brazil; III Universidade Federal do Espírito Santo. Vitória, ES, Brazil

**Keywords:** Tuberculosis, Nursing, Health Policy, Primary Health Care, Public Health., Tuberculosis, Enfermería, Política de Salud, Atención Primaria de Salud, Salud Pública.

## Abstract

**Objective:**

to reflect on the historical role of nurses in tuberculosis prevention in Brazil, considering their inclusion in public policies and advances in Primary Health Care.

**Development:**

based on documents from the World Health Organization, the Ministry of Health, and the literature, this study reflected on nursing care management for people with tuberculosis infection over a century in Brazil.

**Results:**

the role of this professional in the formulation and implementation of preventive public policies for tuberculosis stands out, since 1920, in education, vaccination, home care and, recently, coordinating care management in Primary Care. Competencies have been expanded, supported by resolutions and opinions, consolidating the role of nurses in clinical and managerial practices, particularly in tuberculosis preventive treatment.

**Final considerations:**

the Interministerial Committee and the Healthy Brazil Program are currently reinforcing intersectoral action and the leading role of nurses, strengthening their contribution to the national response against tuberculosis.

## RESUMEN

**Objetivo:**

reflexionar sobre el papel histórico de las enfermeras en la prevención de la tuberculosis en Brasil, considerando su inclusión en las políticas públicas y los avances en la Atención Primaria de Salud.

**Desarrollo:**

con base en documentos de la Organización Mundial de la Salud, el Ministerio de Salud y la literatura, este estudio reflexionó sobre la gestión de la atención de enfermería a personas con infección tuberculosa a lo largo de un siglo en Brasil.

**Resultados:**

se destaca el papel de este profesional en la formulación e implementación de políticas públicas preventivas de la tuberculosis, desde 1920, en la educación, vacunación, atención domiciliaria y, recientemente, coordinando la gestión del cuidado en Atención Primaria. Se han ampliado las competencias, respaldadas por resoluciones y dictámenes, consolidando el rol del personal de enfermería en las prácticas clínicas y de gestión, en particular en el tratamiento preventivo de la tuberculosis.

**Consideraciones finales:**

el Comité Interministerial y el Programa Brasil Saludable están reforzando la acción intersectorial y el liderazgo del personal de enfermería, fortaleciendo su contribución a la respuesta nacional contra la tuberculosis.

## INTRODUCTION

Brazil is considered a priority by the World Health Organization (WHO) as one of the 30 countries with the highest burden of tuberculosis (TB) and TB-HIV co-infection worldwide, accounting for one-third of cases in the Americas. As a signatory to international TB response commitments, Brazil has committed to the WHO’s “End TB” strategies and the 2030 Agenda - Sustainable Development Goals (SDGs). However, there are multiple challenges to achieving the 17 SDGs, including reducing TB, which involves addressing social inequalities that hinder disease control.

Currently, to face this challenge and advance in TB control and elimination as a public health concern, the Ministry of Health (MoH) created the Interministerial Committee for Eliminating Tuberculosis and Other Socially Determined Diseases (In Portuguese, *Comitê Interministerial para a Eliminação da Tuberculose e Outras Doenças Socialmente Determinadas* - CIEDDS)^(1)^ in 2023 with the aim of promoting intersectoral actions for eliminating TB and other socially determined diseases. In 2024, the Healthy Brazil Program^(2)^ was established by Decree 11,908 of February 6, 2024, in line with the objective of eliminating such diseases as a public health concern. This study aimed to reflect on the historical and current role of nurses in TB prevention actions in Brazil, considering their inclusion in public policies and advances in Primary Care.

## DEVELOPMENT

This is a reflective essay, based on WHO documents and strategic TB plans from the Brazilian MoH, on the various processes of nursing care in TB preventive actions over a century, with work in clinical, managerial and prophylactic practices, particularly in TB preventive treatment.

## RESULTS

In relation to TB prevention and control actions, Brazilian nursing has always participated intensely, with emphasis on the beginning of the 20^th^ century, with the Brazilian League Against Tuberculosis (In Portuguese, *Liga Brasileira Contra a Tuberculose*) and, particularly, in the implementation of guidance for affected people and their families, which also occurred in home care, with priority given to health education actions and campaigns^(3)^.

With the Carlos Chagas Reform in 1920, the Brazilian National Department of Public Health and the Tuberculosis Prophylaxis Inspectorate^(3)^ were created, becoming the first state institutions to address TB in the country. The former, in conjunction with the Brazilian Red Cross, offered training courses for health visitors, strengthening families through educational initiatives^(4)^.

Another important activity was nursing participation, from 1927 onwards, in supervising TB control leagues and in oral vaccination of newborns with Bacillus Calmette-Guérin (BCG)^(4)^. In the 1930s, nurses working at the federal level were assigned to collaborate with the federative units in TB actions, in health center organization and in visitor training. The Brazilian National Tuberculosis Service in 1941 strengthened the health, campaigning, and biomedical model. Five years later, the Brazilian National Campaign Against Tuberculosis began using anti-TB drugs in sanatoriums^(4)^, resulting in a reduction in treatment time and a drop in mortality from the disease from 1960 onwards.

Nurses began administering intradermal (ID) BCG vaccine and tuberculin skin test (TST) in this decade, under the guidance of the Brazilian National Tuberculosis Control Campaign Technical Committee. Brazil received assistance from the WHO to standardize the techniques for administering the ID BCG vaccine and TST, enabling to training nurse multipliers^(4)^.

With the deepening of social inequalities, the WHO declared TB a global emergency in 1993. In Brazil, after the implementation of the Family Health Program in 1994, nursing professionals became definitively involved in coordinating care for people with TB and their families, considering the territories where they live, work, and become ill, as well as in implementing managerial actions in Primary Health Care (PHC). In 1996, the Brazilian National Coordination of Sanitary Pulmonology formulated an emergency plan for TB control, emphasizing nursing participation in the active search, diagnosis, and treatment of cases. Additionally, COFEN Resolution 195/1997^(5)^ expanded the role of nurses in PHC, recommending the request for diagnostic tests for TB.

In 2003, the federal government established a specific TB policy, implementing nursing consultations, home visits, an emphasis on person-centered care, and strengthening directly observed treatment (DOT). With the publication of the DOT protocol in 2011, the MoH guides the follow-up treatment of *M. tuberculosis* cases in PHC with nursing participation^(6)^.

Additionally, it recommended notification and recording of treatment for *M. tuberculosis* infection at all levels of care, with management and participation by nursing professionals (Circular Letter 27 of August 13, 2014)^(6)^. To support the implementation of the 2021 Brazilian National Plan to End Tuberculosis, a new protocol expands the role of nurses in PHC, guiding them in *M. tuberculosis* infection identification, diagnosis, and treatment.

Finally, in 2018, the Federal Nursing Council (In Portuguese, *Conselho Federal de Enfermagem* - COFEN) legalized the request for laboratory tests by nurses for diagnosis and prescription of medications for TB treatment (Councilors’ Opinion 180/2018/COFEN)^(7)^. In 2023, Opinion 40/2023^(8)^ strengthened the prescription of medications for *M. tuberculosis* infection treatment and the request for an interferon-gamma release assay. Joint Information Note 04/2024 (COFEN/General Coordination of Tuberculosis and Mycoses)^(9)^ provides technical guidance for nurses to identify, track, and treat cases of *M. tuberculosis* infection in PHC, which constitutes a victory for public health, considering the vulnerability of the most affected populations and the social determination of TB.

The Healthy Brazil Program (2025) strengthens the collaboration between various ministries, social movements, civil society organizations, and other strategic partners, as well as the technical working group of CIEDDS nurses (2024)^(10)^, sealing the political commitment to build a healthier and fairer future for Brazilian families.

Thus, TB prevention has always been a priority for nursing professionals in Brazil. After a century of TB control efforts, these professionals have led strategies to eliminate the disease, despite the enormous challenge of expanding active screening, early diagnosis, encouraging adherence, and increasing preventive treatment.

## FINAL CONSIDERATIONS

The possibility of continuing the intense and significant work of this professional category in combating TB in partnership with the multidisciplinary health team, in addition to the federal guidance that is currently substantially working to reduce social inequalities and the Social Determinants of Health, provides encouragement for achieving TB elimination in the country. Prevention actions are not reduced to the merely technical sphere if we acknowledge that public policies must be uncompromising regarding social inequities.

## Data Availability

The available research data are listed in the body of the article.
