# Supplementary material for: Nurses’ relevance in a century of tuberculosis prevention actions in Brazil
Source: Rev Bras Enferm. 2025 Dec 8;78(6):e20250401. doi: 10.1590/0034-7167-2025-0401 (PMC12707610; doi:10.1590/0034-7167-2025-0401)
Supplement: Translation

# Translation: A relevância dos enfermeiros em um século nas ações de prevenção da tuberculose no Brasil

**DOI:** 10.1590/0034-7167-2025-0401pt

**Authors:** Fernanda Dockhorn Costa Johansen, Maria do Socorro Nantua Evangelista, Maria Rita Bertolozzi, Ethel Leonor Noia Maciel

**Affiliations:** I Ministério da Saúde, Coordenação-Geral de Vigilância da Tuberculose, Micoses Endêmicas e Micobactérias não Tuberculosas. Brasília, DF, Brasil; II Universidade de São Paulo. São Paulo, SP, Brasil; III Universidade Federal do Espírito Santo. Vitória, ES, Brasil

**Keywords:** Tuberculose, Enfermagem, Política de Saúde, Atenção Primária à, Saúde, Saúde Pública.

## Abstract

**Objetivo:**

refletir sobre atuação histórica dos enfermeiros nas ações preventivas da tuberculose no Brasil, considerando sua inserção nas políticas públicas e avanços na Atenção Primária à Saúde.

**Desenvolvimento:**

com base nos documentos da Organização Mundial da Saúde, Ministério da Saúde e literatura, realizou-se uma reflexão sobre a gestão do cuidado dos enfermeiros em pessoas com infecção tuberculosa durante um século no Brasil.

**Resultados:**

destaca-se o papel desse profissional na formulação e execução de políticas públicas preventivas para tuberculose, desde 1920, na educação, vacinação, assistência domiciliar e, recentemente, coordenando a gestão do cuidado na Atenção Primária. Ampliaram-se competências respaldada por resoluções e pareceres, consolidando a atuação do enfermeiro nas práticas clínicas e gerenciais, particularmente no tratamento preventivo da tuberculose.

**Considerações finais:**

o Comitê Interministerial e o Programa Brasil Saudável reforçam atualmente a atuação intersetorial e o protagonismo dos enfermeiros, fortalecendo sua contribuição na resposta nacional contra tuberculose.

## INTRODUÇÃO

O Brasil é considerado prioritário pela Organização Mundial da Saúde (OMS), por ser um dos 30 países com maior carga de tuberculose (TB) e de coinfecção TB-HIV no mundo, concentrando um terço dos casos das Américas. Como signatário dos compromissos internacionais de resposta à TB, o Brasil se comprometeu com as estratégias “*End TB*” da OMS e a Agenda 2030 - Objetivos de Desenvolvimento Sustentável (ODS). Entretanto, múltiplos são os desafios para o cumprimento dos 17 ODS, e entre eles, está a redução da TB, que envolve o enfrentamento das desigualdades sociais dificultando o controle da doença.

Atualmente, para enfrentar esse desafio e avançar no controle e eliminação da TB como problema de saúde pública, foi criado pelo Ministério da Saúde (MS), em 2023, o Comitê Interministerial para a Eliminação da Tuberculose e Outras Doenças Socialmente Determinadas (CIEDDS)^(1)^, no intuito de promover ações intersetoriais para eliminação da TB e demais doenças socialmente determinadas. Em 2024, foi instituído pelo Decreto nº 11.908, de 6 de fevereiro de 2024, o Programa Brasil Saudável^(2)^, alinhado ao objetivo de eliminar tais doenças como problema de saúde pública. O estudo objetiva refletir sobre a atuação histórica e atual dos enfermeiros nas ações preventivas da TB no Brasil, considerando sua inserção nas políticas públicas e avanços na Atenção Primária.

## DESENVOLVIMENTO

Trata-se de ensaio do tipo reflexivo, com base nos documentos da OMS e planos estratégicos de TB do MS do Brasil, sobre os diversos processos de cuidar dos enfermeiros nas ações preventivas da TB em um século, com atuação nas práticas clínicas, gerenciais e profiláticas, particularmente no tratamento preventivo da TB.

## RESULTADOS

No tocante às ações de prevenção e controle da TB, a enfermagem brasileira sempre participou intensamente, com destaque para o início do século 20, com a Liga Brasileira Contra a Tuberculose e, particularmente, na efetivação das orientações junto às pessoas acometidas e suas famílias, o que ocorria também na assistência domiciliária, com prioridade para as ações de educação sanitária e campanhas^(3)^.

Com a Reforma Carlos Chagas, em 1920, foram criados o Departamento Nacional de Saúde Pública e a Inspetoria de Profilaxia da Tuberculose^(3)^, que se constituíram como as primeiras instituições estatais a se ocuparem com a TB no país. O primeiro, em conjunto com a Cruz Vermelha Brasileira, promovia cursos para a capacitação de visitadoras sanitárias, fortalecendo a família com ações educativas^(4)^.

Outra importante atividade foi a participação de enfermeiros, a partir de 1927, na supervisão das ligas de controle da TB e na vacinação oral em recém-nascidos com o Bacilo de Calmette-Guérin (BCG)^(4)^. Na década de 30, enfermeiros que atuavam no âmbito federal foram designados para colaborar com as unidades federativas nas ações da TB, na organização dos centros de saúde e no treinamento de visitadoras. Com o Serviço Nacional de Tuberculose, em 1941, verificou-se o fortalecimento do modelo sanitarista, campanhista e biomédico. Cinco anos depois, a Campanha Nacional Contra a Tuberculose passou a utilizar os medicamentos anti-TB nos sanatórios^(4)^, verificando, a partir de 1960, uma redução do tempo de tratamento e uma queda da mortalidade em decorrência da enfermidade.

Data dessa década a participação de enfermeiros na aplicação da vacina BCG intradérmica (ID) e da prova tuberculínica (PT), por orientação da Comissão Técnica da Campanha Nacional de Controle da Tuberculose. Assinala-se que o Brasil recebeu assessoria da OMS para padronizar as técnicas de administração da vacina BCG ID e da PT, possibilitando a capacitação de enfermeiros multiplicadores^(4)^.

Com o aprofundamento das desigualdades sociais, em 1993, a OMS declarou a TB como emergência mundial. No Brasil, após a implantação do Programa Saúde da Família, em 1994, o profissional de enfermagem se engajou definitivamente na coordenação do cuidado à pessoa com TB e sua família, considerando o território onde vivem, trabalham e adoecem, assim como na efetivação das ações de caráter gerencial da Atenção Primária à Saúde (APS). Em 1996, a Coordenação Nacional de Pneumologia Sanitária formulou um plano emergencial para controle da TB, enfatizando a participação dos profissionais de enfermagem na busca ativa, diagnóstico e tratamento dos casos. Adicionalmente, a Resolução COFEN n^o^ 195/1997^(5)^ ampliou a atuação dos enfermeiros na APS, recomendando a solicitação de exames diagnósticos para TB.

Em 2003, o governo federal estabeleceu uma política específica para TB, com implementação da consulta de enfermagem, realização de visitas domiciliárias, ênfase no cuidado centrado na pessoa e fortalecimento do tratamento diretamente observado (TDO). Com a publicação do protocolo do TDO, em 2011, o MS orienta o seguimento do tratamento dos casos da infecção pelo *M. tuberculosis* na APS com participação da enfermagem^(6)^.

Adicionalmente, recomendou a notificação e o registro do tratamento da infecção por *M. tuberculosis* em todos os níveis de assistência, com gestão e participação dos profissionais de enfermagem (Ofício Circular nº 27, de 13/08/2014)^(6)^. Para dar suporte à implantação do Plano Nacional pelo Fim da Tuberculose de 2021, um novo protocolo amplia o papel dos enfermeiros na APS, orientando-os na identificação, diagnóstico e tratamento da infecção pelo *M. tuberculosis*.

Finalmente, em 2018, o Conselho Federal de Enfermagem (COFEN) legalizou a solicitação de exames laboratoriais por enfermeiros para o diagnóstico e prescrição de medicamentos para o tratamento da TB (Parecer de Conselheiros nº 180/2018/COFEN)^(7)^. No ano de 2023, o Parecer nº 40/2023^(8)^ fortaleceu a prescrição de medicamentos para o tratamento da infecção pelo *M. tuberculosis* e a solicitação do *interferon-gamma release assay*. A Nota Informativa Conjunta nº 04/2024 (COFEN/Coordenação Geral da Tuberculose e Micoses)^(9)^ faz orientações técnicas para enfermeiros identificar, rastrear e tratar os casos de infecção pelo *M. tuberculosis* na APS, o que se constitui em vitória para a saúde pública, considerando a vulnerabilidade das populações mais acometidas e a determinação social da TB.

Com o Programa Brasil Saudável (2025), reforça-se a união dos diversos ministérios, movimentos sociais, organizações da sociedade civil, juntamente com outros parceiros estratégicos e o grupo técnico de trabalho de enfermeiros do CIEDDS (2024)^(10)^, selando o compromisso político de construir um futuro mais saudável e justo para as famílias brasileiras.

Neste sentido, as ações preventivas da TB sempre se constituíram prioridade no trabalho dos profissionais de enfermagem no Brasil. Após um século de ações de controle da TB, esses trabalhadores têm protagonizado estratégias para a eliminação da doença, ainda que diante do gigantesco desafio de ampliação da busca ativa, do diagnóstico precoce, do estímulo à adesão e do incremento do tratamento preventivo.

## CONSIDERAÇÕES FINAIS

A possibilidade de dar continuidade ao intenso e significativo trabalho dessa categoria profissional no enfrentamento da TB em parceria com a equipe multiprofissional de saúde, além da orientação federal que vem, no atual momento, empreendendo substantivamente na redução das desigualdades sociais e dos Determinantes Sociais da Saúde, proporciona um alento para a conquista da eliminação da TB no país. As ações de prevenção não se reduzem à esfera meramente técnica se admitirmos que as políticas públicas devem ser intransigentes com as inequidades sociais.

## Data Availability

Os dados de pesquisa disponíveis estão no corpo do artigo.
